# Lymphocyte activation gene-3-associated protein networks are associated with HDL-cholesterol and mortality in the Trans-omics for Precision Medicine program

**DOI:** 10.1038/s42003-022-03304-0

**Published:** 2022-05-02

**Authors:** Ani Manichaikul, Honghuang Lin, Chansuk Kang, Chaojie Yang, Stephen S. Rich, Kent D. Taylor, Xiuqing Guo, Jerome I. Rotter, W. Craig Johnson, Elaine Cornell, Russell P. Tracy, J. Peter Durda, Yongmei Liu, Ramachandran S. Vasan, L. Adrienne Cupples, Robert E. Gerszten, Clary B. Clish, Deepti Jain, Matthew P. Conomos, Thomas Blackwell, George J. Papanicolaou, Annabelle Rodriguez

**Affiliations:** 1 Center for Public Heath Genomics, University of Virginia, Charlottesville, VA USA; 2 Department of Medicine, University of Massachusetts Medical School, Worcester, MA USA; 3 The Institute for Translational Genomics and Population Sciences, Department of Pediatrics, The Lundquist Institute for Biomedical Innovation at Harbor-UCLA Medical Center, Torrance, CA USA; 4 University of Washington, Seattle, WA USA; 5 University of Vermont, Burlington, VT USA; 6 Duke University, Chapel Hill, NC USA; 7 Department of Biostatistics, Boston University School of Public Health, Boston, MA USA; 8 Department of Medicine, Beth Israel Deaconess Medical Center, Boston, MA USA; 9 Metabolite Profiling, Broad Institute, Cambridge, MA USA; 10 University of Michigan, Ann Arbor, MI USA; 11 National Institutes of Health, Bethesda, MD USA; 12 Center for Vascular Biology, University of Connecticut Health, Farmington, CT USA

**Keywords:** Cardiovascular biology, Genetic association study

## Abstract

Deficiency of the immune checkpoint lymphocyte activation gene-3 (LAG3) protein is significantly associated with both elevated HDL-cholesterol (HDL-C) and myocardial infarction risk. We determined the association of genetic variants within ±500 kb of *LAG3* with plasma LAG3 and defined LAG3-associated plasma proteins with HDL-C and clinical outcomes. Whole genome sequencing and plasma proteomics were obtained from the Multi-Ethnic Study of Atherosclerosis (MESA) and the Framingham Heart Study (FHS) cohorts as part of the Trans-Omics for Precision Medicine program. In situ Hi-C chromatin capture was performed in EBV-transformed cell lines isolated from four MESA participants. Genetic association analyses were performed in MESA using multivariate regression models, with validation in FHS. A LAG3-associated protein network was tested for association with HDL-C, coronary heart disease, and all-cause mortality. We identify an association between the *LAG3* rs3782735 variant and plasma LAG3 protein. Proteomics analysis reveals 183 proteins significantly associated with LAG3 with four proteins associated with HDL-C. Four proteins discovered for association with all-cause mortality in FHS shows nominal associations in MESA. Chromatin capture analysis reveals significant *cis* interactions between *LAG3* and *C1S, LRIG3, TNFRSF1A*, and *trans* interactions between *LAG3* and *B2M*. A LAG3-associated protein network has significant associations with HDL-C and mortality.

## Abstract (web summary)

Rodriguez et al. use whole genome sequencing and plasma proteomics from the Multi-Ethnic Study of Atherosclerosis (MESA) and the Framingham Heart Study (FHS) cohorts of the Trans-Omics for Precision Medicine program and perform in situ Hi-C chromatin capture in cell lines isolated from four MESA participants. They demonstrate that lymphocyte activation gene-3 protein networks are associated with HDL-cholesterol and mortality, which could guide the development of precision medicine.

## Introduction

Cardiovascular disease (CVD) remains the leading cause of mortality despite effective low-density lipoprotein cholesterol (LDL-C) lowering therapies^1–3^. In addition to LDL-C, other lipid CVD risk factors include high-density lipoprotein cholesterol (HDL-C) and triglycerides^4–6^. The causal role of LDL-C in atherosclerosis is now established while that for HDL-C per se is in doubt^7–12^.

A recent review on the topic of high HDL-C paradox examined findings from two large observational studies (Cardiovascular Health in Ambulatory Care Research Team [CANHEART] and Copenhagen City Heart Study and the Copenhagen General Population Study [Copenhagen Heart Studies]) that identified a U-shaped relationship between mortality and HDL-C showing increased mortality risk with low HDL-C but also with high HDL-C levels^13^. Voight et al^12^. showed that in a mendelian randomization study, using either a single nucleotide polymorphism (SNP) for the endothelial lipase gene (*LIPG*) or a genetic score encompassing fourteen common SNPs, high HDL-C levels did not predict lower myocardial infarction (MI) risk. The study concluded that selected genetic variants associated with higher HDL-C levels were not causally associated with MI risk. There have now been a number of randomized clinical trials showing that pharmacologic inhibition of cholesteryl ester transfer protein did not reduce or minimally reduced MI risk despite the effect of significantly raising HDL-C levels from baseline values^7,10,14^.

Candidate gene approaches have shown that common and rare genetic variants within the HDL receptor, scavenger receptor class B type I (*SCARB1*) gene, are significantly associated with increased CVD risk, contributing to the concept of high HDL-C paradox^15–19^. In CARDIoGRAM, a common variant within *SCARB1*, rs10846744 with effect allele C that resides within an enhancer region in the first intron of the gene, is significantly associated with prevalent CVD^20^. A number of experimental approaches have been used to examine the effects of rs10846744 on distally regulating neighboring genes on chromosome 12 (wherein *SCARB1* is located) with a novel physical interaction between *SCARB1* and the immune checkpoint molecule lymphocyte activation gene-3 (*LAG3* gene) and effects on the LAG3 protein having been shown^15^.

LAG3 is a member of the IgG superfamily and is an important immune checkpoint molecule in regulating further activation of T effector cells^21^. The prevailing paradigm is that the extracellular domain of LAG3 on T cells binds with high avidity to a select region on MHC-II molecules on antigen presenting cells to suppress further activation of T cells and regulate T cell homeostasis^22^. In humans, the *LAG3* gene resides on the short arm of chromosome 12 (12p13.32) and is within 8.4 kB of *CD4*^23^. LAG3 is expressed in B cells, T cells, NK lymphocytes, monocytes, and dendritic cells and its distribution is approximately 50% intracellular and 50% on the cell surface^24–26^. Activation of these cells promotes transit of intracellular LAG3 to the cell surface, where extracellular LAG3 is then subject to cleavage by ADAM10 and ADAM17 metalloproteases, resulting in soluble LAG3 (sLAG3)^27^. In addition to transmembrane LAG3 binding to MHC class II to limit effector T cell expansion, in vitro studies have demonstrated that sLAG3 also binds to MHC class II and regulates CD4-driven signaling pathways^28^. A subset of Tregs (alternative Tr1 Tregs) has been characterized using flow cytometric LAG3 expression, with these cells being a major source of secretion of the immunosuppressive interleukin 10 (IL-10) cytokine^29–32^. Zhu et al.^33^ observed that in patients with documented coronary artery disease there was a significantly lower expression by flow cytometry of these CD49b^+^ LAG3^+^ Tr1 Tregs cells compared with control subjects. Our results and that of Zhu et al^33^. are consistent that humans with LAG3 deficiency are at increased risk for CVD and have lower circulating IL-10 levels.

In participants of the Multi-Ethnic Study of Atherosclerosis (MESA), we observed that deficiency of plasma or soluble LAG3 protein was significantly associated with elevated HDL-C levels, and paradoxically with increased risk of CVD^15^. We also reported that age, smoking, lipid medications, and the *SCARB1* rs10846744 were independent predictors of plasma LAG3 levels^15^. In MESA and the Framingham Heart Study (FHS), we have now directly examined the region surrounding the *LAG3* locus to identify common and rare variants associated with plasma LAG3 and HDL-C concentrations, and with clinical outcomes. Additionally, in adjusted models, we examined the association of LAG3 co-expressed proteins and their association with HDL-C, coronary heart disease (CHD) and all-cause mortality. We have now identified a common noncoding variant within the *LAG3* gene that is significantly associated with plasma LAG3, and a number of LAG3 co-expressed proteins that are significantly associated with LAG3 protein, HDL-C, and all-cause mortality.

## Results

### Characteristics of the MESA and FHS participants

The median baseline age of participants from MESA (Exam 1) was 61 years (with an interquartile range of 53.0–69.0 years); 50.7 % were women; and self-reported race/ethnic distribution was 41.5% White, 23.2% Hispanic, 22.8% African-American, and 12.5% Chinese-American (Table 1). In FHS (Offspring Exam 5), the median age of participants was 55 years (with an interquartile range of 47–63 years); 53.5% were women; and were 100% White participants (Table 2).

**Table 1:** Demographic characterization of the MESA participants across race/ethnic groups.

Participant characteristics	Pooled	White	Chinese	African-American	Hispanic
No. subjects	3867	1573	467	876	887
Women	1960 (50.7)	782 (49.7)	231 (49.5)	456 (52.1)	447 (50.4)
Age, years	61 (53,69)	61 (53,69)	61 (52,69)	61 (53,68)	60 (52,68)
Current smoke (yes/no)	462 (12.0)	172 (10.9)	28 (6.0)	151 (17.2)	106 (12.0)
Lipid medication (yes/no)	620 (16.0)	274 (17.4)	73 (15.6)	129 (14.7)	126 (14.2)
BMI, kg/m^2^	27.5 [24.5, 30.9]	27.2 [24.3, 30.3]	23.9 [22.0, 26.1]	29.2 [26.3, 33.2]	28.6 [26.0, 31.7]
fasting glucose (mg/dl)	89 [82,98]	87 [81,94]	91 [85, 101]	91 [83, 101]	92 [84, 103]
Systolic blood pressure (SBP) (mmHg)	122 [111, 138]	120 [110, 135]	120 [108, 137]	129 [116, 142]	122 [111, 139]
Diastolic blood pressure (DBP) (mmHg)	72 [65,79]	71 [64,78]	72 [66,78]	74 [68,81]	72 [65,79]
HDL-C, mg/dl	48 (40,59) (*n* = 3865)	50 (41,61) (*n* = 1572)	48 (40,55) (*n* = 467)	50 (41,60) (*n* = 875)	45 (39,54) (*n* = 887)
LDL-C, mg/dl	116 [96, 136] (*n* = 3819)	115 [96, 136] (*n* = 1555)	113 [96, 131] (*n* = 467)	117 [96, 136] (*n* = 872)	119 [99, 138] (*n* = 869)
Triglycerides, mg/dl	113 [78, 163] (*n* = 3867)	113 [77, 165] (*n* = 1573)	128 [87, 170] (*n* = 467)	90 [66, 121] (*n* = 876)	135 [97, 191] (*n* = 887)
LAG3 protein levels (Ex 1), RFU	6420.8 [5422.5, 7773.1] (*n* = 833)	6401.1 [5360.9, 7802.6] (*n* = 352)	7709.4 [6432.2, 9100.4] (*n* = 62)	6203.7 [5194.9, 7106.0] (*n* = 154)	6388.1 [5512.6, 7533.3] (*n* = 248)
LAG3 protein levels (pg/ml) (Ex 2)	496 [231, 1340] (*n* = 3612)	447 [206, 1144] (*n* = 1526)	674 [322, 1603] (*n* = 462)	536 [202, 2461] (*n* = 881)	505 [261, 1168] (*n* = 847)
Presence of calcium indicator (yes/no)	1806 (47.3) (*n* = 3819)	819 (52.7) (*n* = 1555)	221 (47.3) (*n* = 467)	358 (41.1) (*n* = 872)	375 (43.2) (*n* = 869)
Agatston calcium score (among those with CAC > 0)	77.9 [18.9, 274.2] (*n* = 1806)	99.7 [20.2, 328.8] (*n* = 819)	67.1 [25.5, 221.5] (*n* = 221)	53.1 [17.2, 232.5] (*n* = 358)	73.8 [18.9, 248.0] (*n* = 375)
Common carotid intimal-medical thickness (mm)	0.84 [0.73, 0.96] (*n* = 3804)	0.83 [0.72, 0.96] (*n* = 1549)	0.80 [0.70, 0.91] (*n* = 467)	0.88 [0.76, 1.00] (*n* = 866)	0.82 [0.73, 0.93] (*n* = 866)
Internal carotid intimal-medical thickness (mm)	0.84 [0.68, 1.20] (*n* = 3746)	0.87 [0.71, 1.31] (*n* = 1532)	0.73 [0.60, 0.88] (n = 462)	0.85 [0.67, 1.28] (*n* = 850)	0.82 [0.66, 1.15] (*n* = 847)
Ankle-brachial index (ABI)	1.13 [1.06, 1.19] (*n* = 3788)	1.14 [1.07, 1.20] (*n* = 1540)	1.12 [1.07, 1.18] (*n* = 466)	1.10 [1.03, 1.16] (*n* = 863)	1.14 [1.08, 1.20] (*n* = 865)
Stroke (yes/no)	179 (0.047) (*n* = 3816)	72 (0.046) (*n* = 1553)	17 (0.036) (*n* = 467)	35 (0.040) (*n* = 872)	54 (0.062) (*n* = 868)
Myocardial infarction (MI) (yes/no)	188 (0.049) (*n* = 3816)	84 (0.054) (*n* = 1553)	17 (0.036) (*n* = 467)	34 (0.039) (*n* = 872)	51 (0.059) (*n* = 868)
Follow-up time (days)	5539 [5317, 5745]	5610 [5357, 5791]	5538 [5311, 5745]	5456 [5280, 5658]	5509 [5289, 5738]

Data are presented as *n* (%) for binary measures or median [Interquartile range (IQR)] for continuous measure. *N* = number of participants in each variable. Descriptive statistics for covariates and phenotypes are reported based on the baseline examination (Exam 1), except where noted otherwise. Descriptive statistics for events are presented based on MESA adjudication through the year 2016. Summary statistics are reported based on the subset of samples included in genetic analyses. SOMAscan proteomics, RFU = relative fluorescent units.

**Table 2:** Demographic characterization of FHS participants.

Participant characteristics	FHS
No. subjects	1913
Women	1024 (53.5)
Age, years	55 (47,63)
HDL-C, mg/dl	48 (39,59) (*n* = 1907)
LDL-C, mg/dl	125 [104, 146] (*n* = 1855)
Triglycerides, mg/dl	121 [85, 179] (*n* = 1913)
BMI, kg/m^2^	26.7 [24.0, 29.9] (*n* = 1910)
fasting glucose	95 [89, 104] (*n* = 1897)
Systolic blood pressure (SBP), mmHg	124 [113, 138] (*n* = 1913)
Diastolic blood pressure (DBP), mmHg	74 [68,81] (*n* = 1913)
Current smoke (yes/no)	370 (19.3) (*n* = 1912)
Lipid medication (yes/no)	142 (7.4) (*n* = 1913)
LAG3 protein levels (Ex 5), RFU	4764 [3635, 6204] (*n* = 1913)
Presence of calcium indicator (yes/no)	NA
Agatston calcium score, phantom-adjusted	NA
Common carotid intimal-medical thickness (mm)	NA
Internal carotid intimal-medical thickness (mm)	NA
Ankle-brachial index (ABI) (Gen 3)	1.23 [1.18, 1.28] (*n* = 2609)
Myocardial infarction (MI) (yes/no)	97 (5.1)
Follow-up time (days)	7039 days

Data are presented as *n* (%) for binary measures or median [Interquartile range (IQR)] for continuous measure. *N* = number of participants in each variable. Exam = FHS proteomics data for Examination 5.

### Genetic associations with phenotypic traits

#### Discovery study in MESA

**Fig. 1:**
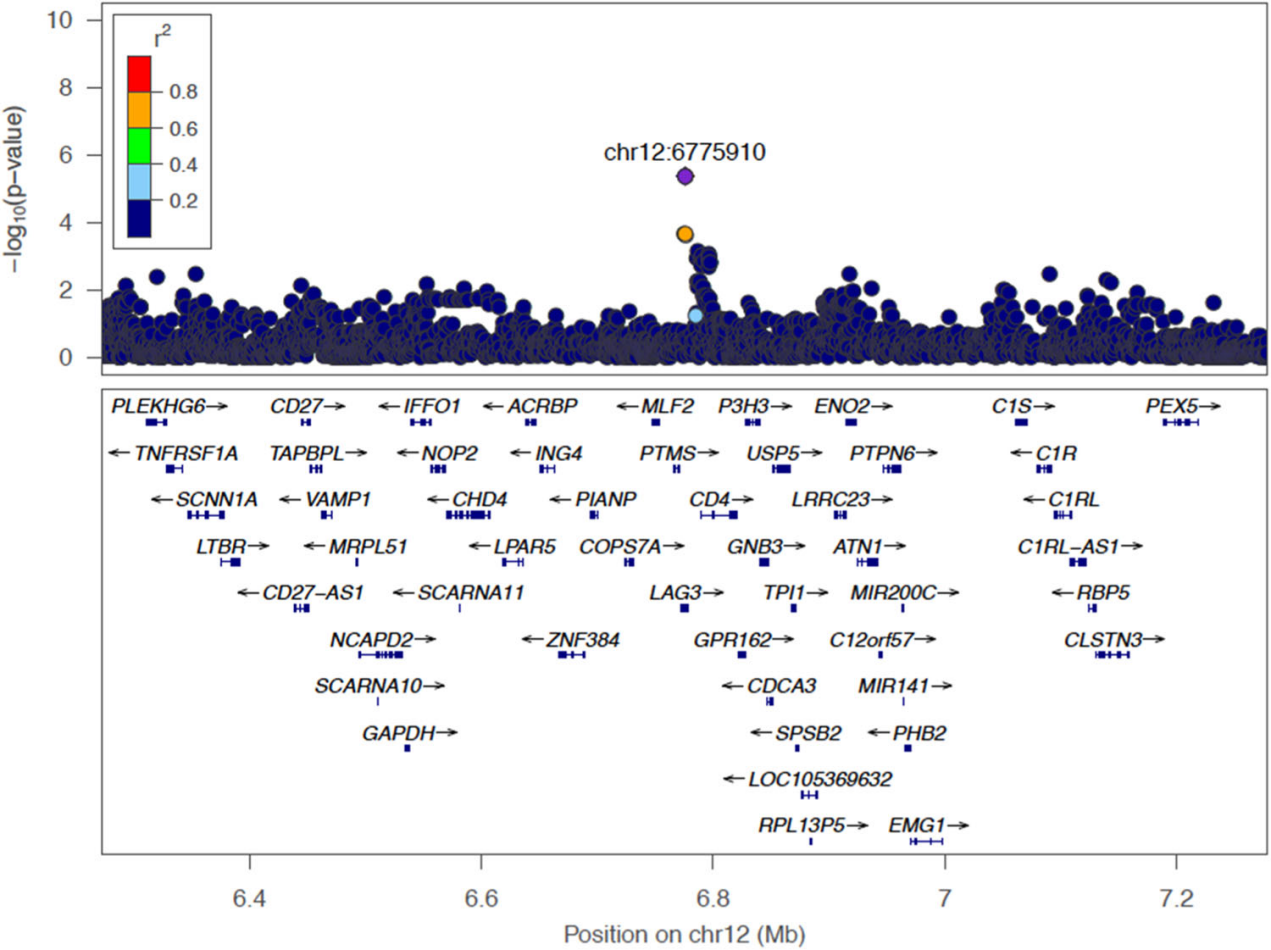
Regional association plots for statistically significant genetic association study region based on meta-analysis results of MESA on LAG3 protein levels in Exam 1. The plot presents results for the index variant rs3782735 at chr12:6775910 +/- 250 kb, with linkage disequilibrium determined using the multi-ethnic TOPMed WGS data from MESA.

Meta-analysis identified one significant genetic variant within the *LAG3* region at FDR < 0.05 (based on a Z-test from meta-analysis for the regression coefficient; Table 3, Fig. 1). The common *LAG3* variant rs3782735 (chr12:6775910) effect allele G was positively associated with LAG3 protein levels (Beta = 0.24 for the additive genetic effect on inverse normal transformed protein level; 95% confidence interval [CI] = 0.14–0.34; Z-stat = 4.8; *P*-value = 4.28 × 10^−6^; FDR = 0.014) at Exam 1. In race/ethnic-stratified analysis, rs3782735 showed statistically significant association in the larger White group only (FDR = 0.003) (associations of rs3782735 with other phenotypic traits shown in Supplementary Data 1). The common *C1S* variant rs7970720 (chr12:7048232) effect allele G was negatively associated with peripheral vascular disease as measured by ABI at Exam 5 (FDR = 0.016) (Supplementary Fig. 1). In race/ethnic-stratified analysis, rs7970720 was statistically significant in the larger White group only (FDR = 0.0004).

#### Validation study in FHS

In FHS, rs3782735 was significantly associated with LAG3 protein levels (Beta = 0.105 for the additive genetic effect on log-transformed protein level; 95% CI = 0.018-0.192; *t*-stat = 2.38; *P*-value = 0.018 based on a two-sided *t*-test for the coefficient from regression with *n* = 1007), but rs7970720 did not demonstrate association with ABI (*P*-value > 0.05).

#### Discovery study in FHS

**Fig. 2:**
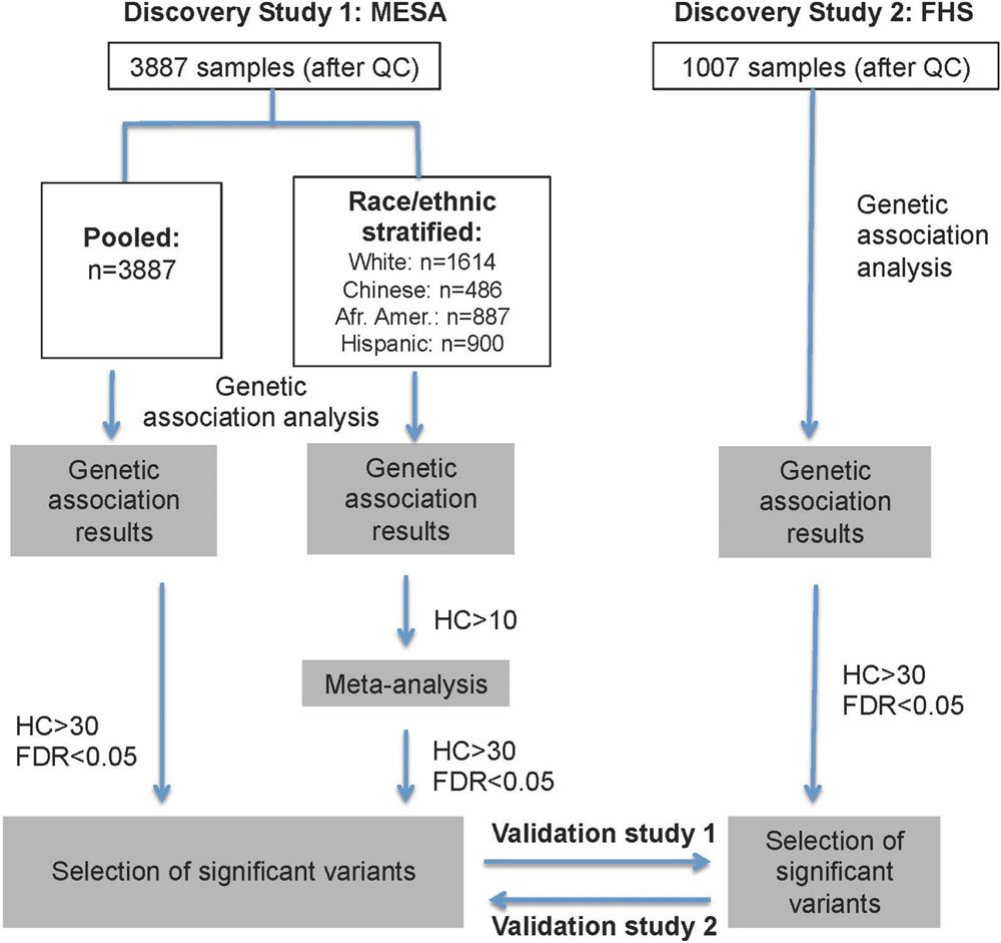
Study design for discovery/validation of genetic association analysis of LAG3 protein levels in MESA and FHS. Abbreviations: Afr. Amer. = African American; HC = heterozygosity count; FDR = False discovery rate.

In the FHS cohort, we did not identify any SNPs that were significantly associated with LAG3 protein levels after adjusting for multiple testing (FDR < 0.05 based on a two-sided *t*-test for each coefficient from regression with *n* = 1007). Thus, there was no follow-up of FHS-based findings in MESA (Fig. 2, validation study 2).

**Table 3:** Genetic association analysis in MESA.

Trait	Chr:Pos (Build 38) Ref/effect allele (rsid)	Group	Exam	N	Beta	SE	EAF	*P*-Value	FDR	HC
LAG3 protein levels	12:6775910 G/A (rs3782735)	White	1	352	0.34	0.07	0.39	1.39E-06	**0.003**	169
		Chinese-American	1	62	0.19	0.20	0.45	0.345	0.925	34
		African-American	1	153	0.09	0.15	0.22	0.545	0.971	48
		Hispanic	1	248	0.10	0.10	0.37	0.312	0.997	125
		Meta-Analysis	1	815	0.24	0.05	0.64	4.28E-06	**0.014**	376
Ankle-brachial index	12:7048232 G/T (rs7970720)	White	5	1330	−0.03	0.01	0.18	5.59E-07	**0.0004**	394
		Chinese-American	5	369	−0.02	0.01	0.08	0.200	0.999	59
		African-American	5	720	−0.01	0.01	0.20	0.328	0.849	219
		Hispanic	5	721	−0.003	0.01	0.11	0.825	0.997	138
		Meta-Analysis	5	3140	−0.02	0.01	0.84	5.81E-06	**0.016**	810

*N* = number of participants. Ref=reference or non-effect allele. EAF = effect allele frequency. HC = heterozygosity count. Numbers in the FDR column that are bolded are done to improve the readability of the table.

Race/ethnic-specific *P*-values are based on two-sided *t*-tests for regression coefficients with covariate adjustment for age, sex, study site, principal components (PCs) of ancestry (2 PCs for White, 1 PC for Chinese, 1 for African-American, and 3 for Hispanic, and 5 PCs for race/ethnic pooled analyses), self-reported race/ethnicity (pooled-group analysis only), HDL-C, LDL-C, triglycerides, body mass index (BMI), fasting glucose, SBP, diastolic blood pressure (DBP), current smoking, former smoking, and lipid medication use. *P*-values combined across race/ethnic groups are based on Z-tests for fixed-effect meta-analysis across the four groups.

### LAG3 protein association analysis

#### Discovery study in MESA and validation in FHS

Next, we examined the extent of plasma LAG3-associated protein networks by performing a multivariate linear regression analysis with LAG3 as the outcome. The model examined association of each protein measure with LAG3 under covariate adjustment for age, sex, and PCs of ancestry. LAG3 protein levels in MESA were associated with 669 other protein measures at Exam 1 and 968 proteins at Exam 5 (FDR < 0.05 based on a two-sided *t*-test for the coefficients from regression with *n* = 938 and *n* = 929 at MESA Exams 1 and 5, respectively). Among these, 603 proteins were identified at FDR < 0.05 at both MESA Exams 1 and 5 (Supplementary Data 2), and these proteins were carried forward for validation in FHS. Among these 603 protein measures, 254 demonstrated significant association with LAG3 protein levels in FHS after adjusting for multiple testing (*P* < 0.05/603 = 8.3 × 10^−5^ based on a two-sided *t*-test for the coefficient from regression with *n* = 1913; Supplementary Data 3).

#### Discovery study in FHS and validation in MESA

We identified 657 protein measures that were associated with LAG3 protein levels in the FHS proteomics data (FDR < 0.05 based on a two-sided *t*-test for the regression coefficient; Supplementary Data 4). Among them, 287 and 431 demonstrated significant associations with LAG3 protein levels in MESA Exams 1 and 5, respectively, after adjusting for multiple testing (*P* < 0.05/657 = 7.6 × 10^−5^ based on a two-sided *t*-test for the regression coefficient). Among the associations identified for Exams 1 or 5, 261 of the identified protein measures overlapped with Bonferroni-corrected validation evidence at both MESA Exams 1 and 5 (Supplementary Data 5). Of these, 131 proteins were negatively associated with LAG3 while 130 were positively associated with LAG3.

#### Overlap of proteins discovered and validated in both MESA and FHS

We identified 183 LAG3 associated proteins that overlapped among the validated lists of associated proteins in FHS, MESA Exam 1, and MESA Exam 5 (Supplementary Data 6). Pathway analysis of the 183 overlapped proteins using GeneAnalytics^TM^ algorithms revealed sixty-four significant high disease score matches with malignancy, autoimmune disease, neurological disorders, and vascular disease and one hundred fifty-one high scores in the innate immune system pathway (Supplementary Data 7).

### LAG3 protein network and association with HDL-C

**Fig. 3:**
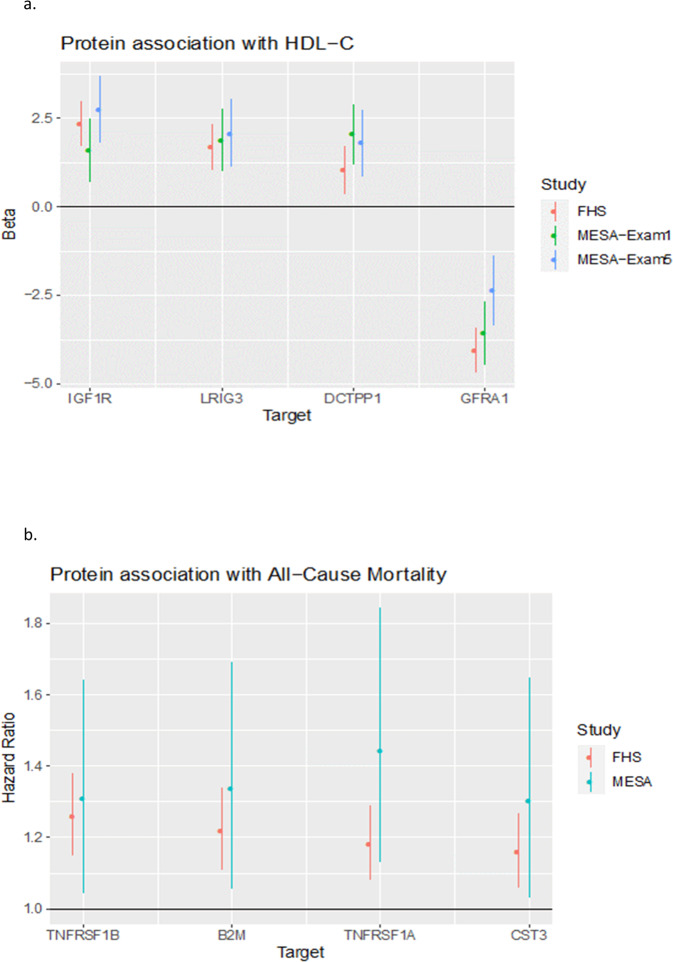
LAG3 related proteins with (a) HDL-C associations discovered and validated in both MESA and FHS, and (b) all-cause mortality associations discovered in FHS and with nominal support from MESA. Plots show estimated effects and 95% confidence limits. Analyses were performed with inverse normal transformed protein levels in MESA and log-transformed protein levels in FHS. **a** Association of HDL-C with protein levels was examined by linear regression with covariate adjustment for age, sex, study site (in MESA), race/ethnicity (in MESA), PCs of ancestry, BMI, triglycerides, pack-years of smoking (in MESA), current smoking (in FHS), current alcohol use, LAG3 protein level, plate ID and batch (in FHS). Among the 183 proteins examined for association with HDL-C in both MESA and FHS, four of them were discovered and validated in both cohorts (leucine rich repeats and immunoglobulin like domains 3 [LRIG3], DNV family receptor alpha 1 [GFRA1], insulin like growth factor 1 receptor [IGF1R] and DCTP pyrophosphatase 1 [DCTPP1]). **b** Of the 183 proteins examined for association with CHD and all-cause mortality in MESA, we did not observe any results at FDR < 0.05. In FHS, while there were no associations at FDR < 0.05 for CHD, we observed that 18 of the 183 LAG3-associated proteins demonstrating FDR < 0.05 were significantly associated with all-cause mortality based on two-sided Z-tests for the coefficients from Cox regression with a total sample of *n* = 1913, including 650 events (Supplementary Data 12). In MESA, none of these 18 proteins reached Bonferroni-corrected statistical significance, but four of the 18 proteins showed nominal associations with all-cause mortality (tumor necrosis factor receptor super family 1 A [TNFRSF1A], beta-2-microglobulin [B2M], tumor necrosis factor receptor super family 1B [TNFRSF1B] and cystatin C [CST3]; all *P* < 0.05 based on two-sided Z-tests for the coefficients from Cox regression with *n* = 935 including 95 events. Association of baseline protein levels with all-cause mortality was examined under a Cox proportional hazards model with covariate adjustment for age, sex, race/ethnicity (in MESA), PCs of ancestry, BMI, total cholesterol, HDL-C, SBP, DBP, pack-years of smoking (in MESA), current smoking status (in FHS) and batch (in FHS).

Since we previously observed a significant inverse association between plasma LAG3 and HDL-C^15^, we next examined the association of the LAG3 protein network with HDL-C. Of the overlapped 183 LAG3-associated proteins (Supplementary Data 6), thirteen demonstrated associations with HDL-C in MESA with FDR < 0.05 at both MESA Exams 1 and 5 (based on a two-sided *t*-test for the coefficients from regression with *n* = 787 and *n* = 927 at MESA Exams 1 and 5, respectively; Supplementary Data 8). Among these thirteen HDL-C associated proteins in MESA, six of them demonstrated Bonferroni significant validation in FHS (*P* < 0.05/13 based on a two-sided *t*-test for the coefficient from regression with *n* = 1907, Supplementary Data 9). In FHS, 88 of the overlapped 183 LAG3 associated proteins showed associations with HDL-C at FDR < 0.05 based on a two-sided *t*-test for each coefficient from regression (Supplementary Data 10), and six of these further showed Bonferroni corrected validation in MESA (*P* < 0.05/88) at both Exams 1 and 5 (Supplementary Data 11). Among the 183 proteins examined for association with HDL-C in both MESA and FHS, four of them were discovered and validated in both cohorts (leucine rich repeats and immunoglobulin like domains 3 [LRIG3], DNV family receptor alpha 1 [GFRA1], insulin like growth factor 1 receptor [IGF1R] and DCTP pyrophosphatase 1 [DCTPP1]; Fig. 3a).

### LAG3 protein network and association with clinical outcomes (coronary heart disease and all-cause mortality)

Of the 183 proteins examined for association with CHD and all-cause mortality in MESA, we did not observe any results at FDR < 0.05. In FHS, while there were no associations at FDR < 0.05 for CHD, we observed that 18 of the 183 LAG3-associated proteins demonstrating FDR < 0.05 were significantly associated with all-cause mortality based on two-sided Z-tests for the coefficients from Cox regression with a total sample of *n* = 1913, including 650 events (Supplementary Data 12). In MESA, none of these 18 proteins reached Bonferroni-corrected statistical significance, but four of the 18 proteins showed nominal associations with all-cause mortality (tumor necrosis factor receptor super family 1 A [TNFRSF1A], beta-2-microglobulin [B2M], tumor necrosis factor receptor super family 1B [TNFRSF1B] and cystatin C [CST3]; all *P* < 0.05 based on two-sided Z-tests for the coefficients from Cox regression with *n* = 935 including 95 events; Fig. 3b and Supplementary Data 13).

### Chromatin capture interactions between *LAG3* and loci associated with HDL-C and all-cause mortality

**Fig. 4:**
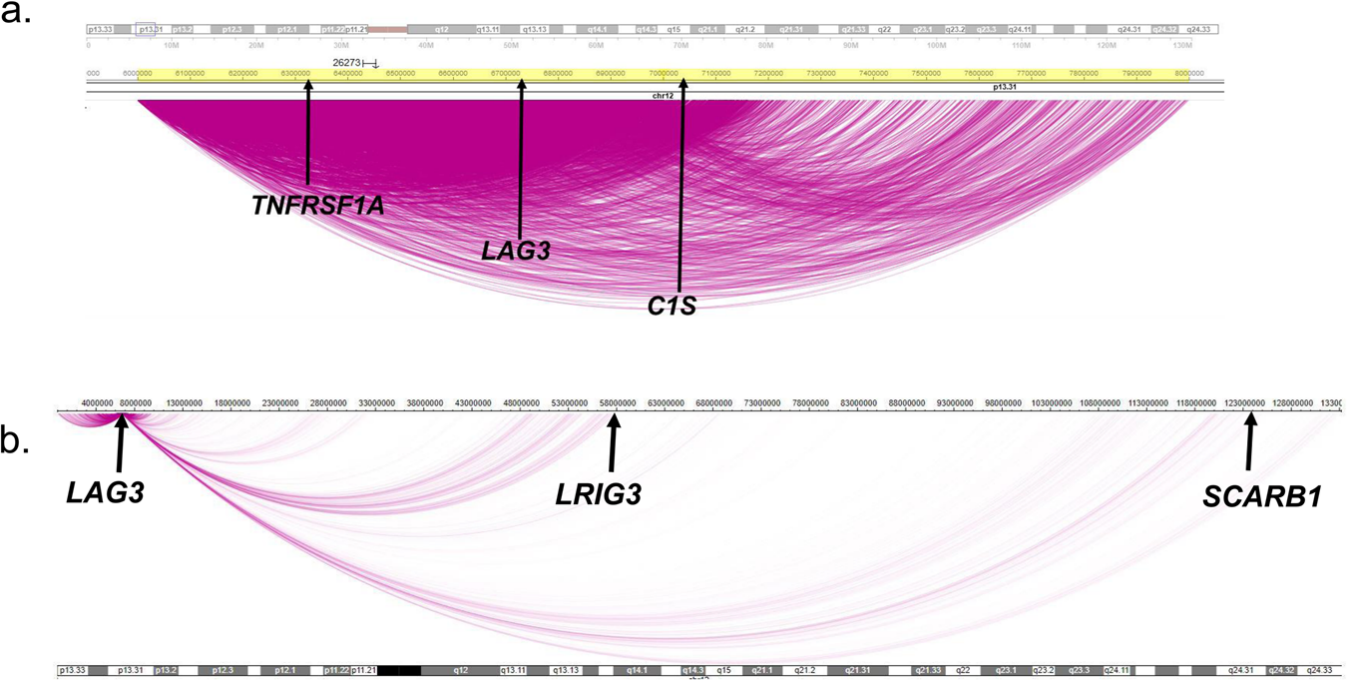
Hi-C near *cis* and *cis* interactions from the *LAG3* locus on chromosome 12: MESA. We performed in situ Hi-C analysis in EBV-transformed B lymphoblasts from two female (one African-American and one Hispanic) MESA carriers homozygous for the *SCARB1* rs10846744 reference G and two female (one African-American and one Hispanic) MESA carriers homozygous for the effect C alleles. The in situ Hi-C analysis was performed as recommended by the 4D Nucleome Consortium using the four base pair cutter *DpnII* restriction enzyme and high read depth next gen sequencing (NGS) to maximize resolution of the high frequency interactions between chromatin contacts (https://www.4dnucleome.org/protocols.html). Each cell library underwent deep NGS at read depths between 1.4–3.3 billion and this was done independently twice as technical replicates for each cell library. Bioinformatic analysis was conducted using Hi-C Pro software with binning of the matrix at different resolutions and iterative correction and eigenvector decompensation normalization of the matrix for each of the four libraries^62,63^. Readouts were all valid paired-end reads and corresponding high frequency contact interaction scores. We then used data generated from the million binning resolution and filtered it based on the *LAG3* chromosomal coordinates (chr12:677250-6778455) using human assembly GRCh38/hg38 (https://genome.ucsc.edu/cgi-bin/hgGateway), which yielded both *cis* (chr12) and *trans* interactions. We set interaction scores at the *LAG3* locus arbitrarily at 1 and then compared interaction scores from direct and indirect *cis* and *trans* interactions. Student t-test was performed with *P* ≤ 0.05 considered statistically significant. The results shown are representative from one of the MESA cell lines, with the schematic representing the near cis (panel **a**) and cis interactions (panel **b**) from the *LAG3* locus on chromosome 12.

In a hypothesis seeking effort, we performed in situ Hi-C chromatin capture to examine possible chromatin high frequency interactions between *LAG3* (chr12: 6772483) and the loci associated with HDL-C (*LRIG3, GFRA1, IGF1R*, and *DCTPP1*) and all-cause mortality (*TNFRSF1A, TNFRSF1B, B2M*, and *CST3*). As a representative example of the Hi-C assays, results from an African-American female MESA participant homozygous for the *SCARB1* rs10846744 effect C allele showed significant *cis* interactions between *LAG3* and *LRIG3* (chr12: 58872149; interaction score [IS] = 17.4; *n* = 22 read pairs; *P* < 0.0001 based on two-sided Student *t*-test) (Fig. 4). No significant *trans* interactions were observed between *LAG3* and *GFRA1* (chr10:116056925)*, IGF1R* (chr15:98648539) or *DCTPP1* (chr16: 30423615).

For loci associated with all-cause mortality, significant *cis* interactions were observed between *LAG3* and *TNFRSF1A* (chr12: 6328757, IS = 3308; *n* = 13932 read pairs; *P* < 0.0001 based on two-sided Student *t*-test). Significant *trans* interactions were observed between *LAG3* and *B2M* (chr15: 44711487, IS = 11.95; *n* = 23 read pairs; *P* < 0.0001 based on two-sided Student *t*-test), but not with *TNFRSF1B* (chr1: 12166948) or *CST3* (chr20: 23626706).

## Discussion

LAG3 is an important immune checkpoint molecule with continued interest in its role as an immuno-oncology therapeutic agent^34^. We were the first to report the association of LAG3 protein deficiency with HDL-C and increased risk of MI, and had identified independent predictors for plasma LAG3 protein^15^. In the present study, we examined the association of variants within ±500 kb of the *LAG3* locus in TOPMed MESA and FHS to identify those significantly associated with plasma LAG3 protein concentrations and clinical outcomes. We identified the *LAG3* rs3782735 variant as significantly associated with plasma LAG3 protein levels, and neighboring *C1S* rs7970720 variant as significantly associated with peripheral vascular disease (ABI) in the MESA population (Supplementary Fig. S1). The *LAG3* rs3782735 is an intronic variant that appears to reside within an enhancer region, has been associated with multiple myeloma risk in women, but has not yet been associated with lipids, CHD or mortality risk^35^.

We next examined the association of LAG3 with plasma proteins in the TOPMed SOMAscan proteomics datasets available in MESA and FHS. We identified 183 LAG3-associated proteins between MESA and FHS, with pathway analysis revealing sixty-four high disease matches in malignancy, autoimmune diseases, neurological disorders, vascular diseases including MI (AKT1, CCL5, CD163, CDH5, CST3, CXCL12, ENG, IL6R, LTA, PF4, PPBP, PSMA6, SELL, TEK, THBS2, TLR4, TNFRSF1A, VCAM1) and heart disease (ANGPT2, CMA1, CST3, CXCL12, DSCAM, ENG, JAG1, NOTCH1, PF4, PPBP, TLR4, TNFRSF1A, TNFRSF1B, VCAM1). Under pathway analysis, we identified high score matches for fluid shear stress and atherosclerosis (AKT3, AKT1, PDGFB, AKT2, BCL2, CDH5, VCAM1, PDGFA, TNFRSF1A). Additionally, when we performed pathway analysis for proteins positively associated with LAG3 we found high score matches for pigment epithelium-derived factor (PEDF) induced signaling while negatively associated proteins had high score matches for innate immune system pathways. These significant LAG3-associated proteins identified in both MESA and FHS provide a framework to examine this network in mediating various inflammatory and non-inflammatory diseases.

Given that plasma LAG3 was shown to be inversely associated with HDL-C, we examined the association of the 183 LAG3-associated overlapping proteins in MESA and FHS with HDL-C using adjusted models as shown in Fig. 3. In MESA and FHS, GFRA1 showed an inverse association with HDL-C, while LRIG3, IGF1R, and DCTPP1 were all positively associated with HDL-C. A PubMed literature search did not reveal a known connection between these four proteins and LAG3 or connections with HDL-C except for LRIG3, suggesting novel findings of these proteins with lipid metabolism. LRIG3 has been shown to be associated with HDL-C in both humans and animals^36–38^. In particular, FHS investigators examined single-locus and epistasis variants on HDL-C and identified a region near *LRIG3* as being significant^36^. In another study in African-American subjects, the region near *LRIG3* was found to be significantly associated with incident congestive heart failure (CHF)^37^. In *Lrig3*^*−/−*^ mice, investigators showed that aging mice demonstrated cardiac hypertrophy and low plasma HDL-C levels^38^.

In MESA plasma LAG3 was shown to be inversely associated with increased MI risk^15^. Therefore, we examined the association of the overlapping 183 proteins with CHD and all-cause mortality in MESA and FHS as shown in Fig. 3. In MESA, there were significant associations of EFNA5, ENG, IL1R1, and NRCAM with CHD risk but the number of events were extremely small and thus caution was taken in interpreting these results. In MESA, we did not observe significant association with all-cause mortality. In FHS, there were 18 proteins significantly associated with all-cause mortality, with 4 proteins nominally associated with mortality in MESA. Abers et al^39^. recently reported the significant independent association of soluble TNFRSF1A with mortality in patients hospitalized with COVID-19. Schnabel et al^40^. reported that TNFRSF1B (also known as TNFR2) was significantly associated with cardiovascular disease and mortality in FHS. Zaghlool et al^41^. performed a step-wise epigenome-wide association analysis with plasma proteomics measured by SOMAscan in two cohorts (Cooperative Health Research in the Region of Augsburg and Qatar Metabolomics Study on Diabetes), and identified nine association pathways. One of these was identified as a chronic inflammatory pathway represented by *NLRC5* and included LAG3, B2M, CD48, CXCL10, FCGR3B, CD163, and CXCL11. We have now identified a LAG3-association network with all-cause mortality that includes B2M (a component of the class I major histocompatibility complex molecule involved in antigen presentation^42^) and CD48 (Supplementary Data 12). We also identified that *LAG3* and *B2M* have significant trans chromatin interactions, which is consistent with the trans contacts between *NLRC5* and *LAG3* and *B2M* reported by Zaghlool et al^41^. Also of importance, these authors identified an inverse association of LAG3 with HDL-C, confirming our initial observation.

Pioneers in the field of 3D chromatin architecture have developed and continue to refine methodologies that evaluate the effect of *cis* and *trans* gene-gene interactions on downstream gene regulation^43–46^. We used Hi-C assays to evaluate unbiased global chromatin interactions (many-to-many) without immunoprecipitation followed by high depth NGS. We then used a number of bioinformatic programs to assess the quality of the technical and biological replicates in order to evaluate the chromatin interactions from the *LAG3* locus^47–49^. In particular, HiCcompare allows statistical analysis of technical and biological replicates for *cis* interactions, while *trans* interactions can be challenging^50^. We observed that *LAG3* has strong chromatin interactions with *C1S, LRIG3, and TNFSRSF1A*, all in close proximity to the *LAG3* locus, which is consistent with observations that chromatin contact interactions are strongest with small differences in genomic distance. While we did observe significant *LAG3* chromatin interactions with *trans* contacts, we want to proceed with caution as the absolute value of the interaction scores were low as well as the number of read pairs. It could be possible there are biological effects with the *LAG3 trans* chromatin contacts but we acknowledge there are post-translational processes that more likely influence LAG3 protein interactions. Nonetheless, the significant *trans* interactions lends support to the hypothesis that *trans* chromatin contacts could explain some of the variance in the LAG3-associated protein network.

In conclusion, we have identified a common *LAG3* variant that is associated with plasma LAG3 protein levels, while adjacent genes in significant chromatin contact with the *LAG3* locus were associated with HDL-C (*LRIG3*) and clinical outcomes such as all-cause mortality (*TNFRSFA1* and *B2M*). This LAG3-associated network analysis identifies novel targets for further study in HDL metabolism, cardiovascular diseases, and all-cause mortality.

## Methods

### Trans-omics for precision medicine (TOPMed)

This study was approved by the TOPMed Publications & Presentations Steering Committees with data access provided by an approved project (#21279) through a database of Genotypes and Phenotypes (dbGaP) application. The study cohorts that comprise this analysis include MESA and FHS.

### Study participants

MESA is a longitudinal study of subclinical CVD and risk factors that predict progression to clinically overt CVD or progression of the subclinical disease. The first clinic visits (Exam 1) occurred from 2000 to 2002 in 6,814 participants recruited from 6 field centers across the United States, and all participants were free of clinical CVD at Exam 1. The self-reported ancestry distribution is approximately 38% White, 28% African-American, 22% Hispanic, and 12% Asian (predominantly of Chinese descent)^51^. MESA has been enhanced by many ancillary studies focused on specific phenotypic and exposure domains. One ancillary study (MESA Family Study) exclusively recruited African-American and Hispanic family members specifically for genetic studies. In contrast, the FHS is a single community-based cohort initiated in 1948. Three generations of participants have been recruited, and the majority of them were white individuals of European ancestry. Participants were invited to attend physical examinations every 2–8 years. The current study was restricted to the Offspring cohort participants who attended their fifth clinical examination cycle during 1991–1995^52^.

All MESA participants provided written informed consent for participation at the six field sites, and FHS participants provided written informed consent for participation at the single site in Framingham, MA. MESA and FHS study protocols were both reviewed and approved by the Institutional Review Boards (IRBs) at each of the participating study sites, as well as at the University of Virginia (AM), Boston University Medical Campus (HL) and the University of Connecticut Health (AR).

### Genotype data

We used the TOPMed Freeze 8 whole genome sequencing (WGS) data, focused on the region within ±500 kb of the *LAG3* gene for examination of our genotype data. TOPMed WGS was conducted at a mean depth of >30X using Illumina HiSeq X Ten instruments. Variant discovery and genotype calling was performed jointly across all studies by the TOPMed Informatics Research Center (IRC) using the GotCloud pipeline. Variant quality control (QC) was performed by the IRC using support vector machine filtering to identify high quality variants. WGS sample QC including sample identity and consent checks was performed by the TOPMed Data Coordinating Center.

### Proteomic data

For MESA Exam 2 (*n* = 5623), LAG3 protein ELISA kits were purchased from RayBiotech, Inc. (Norcross, GA) and LAG3 was measured per the manufacturer’s instructions^15^. Briefly, aliquots of fasting plasma samples stored at −80 °C were thawed, diluted 3-fold, and then 100 µl were used for duplicates per sample for plasma LAG3 measurement. The intra-assay coefficient of variance was <10% and inter-assay CV < 12%. The R^2^ for the standard curve, which was run for each plate, had mean values >0.98. This ELISA has not been validated for clinical use. The single-stranded DNA aptamer-based SOMAscan**™** proteomics platform was used to assay plasma samples from MESA Exams 1 (years 2000–2002) and 5 (years 2010–2012), and from FHS Exam 5 (years 1991–1995). All MESA samples were profiled with Version 1.3k. FHS samples were either profiled with Version 1.1k with 1124 aptamers or with Version 1.3k with 1305 aptamers. All assays were performed using SOMAscan reagents according to the manufacturer’s detailed protocol^53^. The strength of this platform is its ability to measure multiple proteins in a single small aliquot. As reported by SOMALogic, the assay measures >1300 proteins from a small biological sample (150 µl) with low limits of detection and 5% median coefficient of variation^53,54^. This technology uses DNA aptamers (short single-stranded oligonucleotides) that bind to proteins and is quantified by hybridization to custom DNA microarrays. The units are reported as Relative Fluorescent Units (RFU) that are directly proportional to the amount of target protein.

### Phenotype data

Phenotypes of this study were obtained from MESA Exam 1, Exam 2 (years 2002–2004), and Exam 5. The MESA Family Study (years 2004–2006) examination data and event data were combined with MESA Exam 1 data. Fasting blood samples were drawn and processed using a standardized protocol and sent for measurement of lipid levels^55^. LDL-C was calculated using the Friedewald formula. Triglycerides were measured using a glycerol-blanked enzymatic method with the Triglyceride GB reagent on the Roche COBAS FARA centrifugal analyzer. Measures of subclinical atherosclerosis examined included the presence (defined by a cutoff of coronary artery calcium [CAC] > 0) or absence of CAC, calcium Agatston score (phantom-adjusted) and ultrasound measurements of intima-media thickness (IMT) in millimeters (mm) for common and internal carotid IMT, and the ankle brachial index (ABI)^56^. Systolic blood pressure (SBP) measurements in the bilateral brachial, dorsalis pedis, and posterior tibial arteries were obtained in the supine position using a hand-held Doppler instrument with a 5-mHz probe. Cardiovascular events including stroke, MI, CHD and all-cause mortality were adjudicated by a MESA committee of neurologists, cardiologists, and physician epidemiologists, who provided a detailed description of the cardiovascular event adjudication process^57^. In FHS, the Friedewald formula was used to calculate LDL-C, and a modification of the Kessler-Lederer method was used to measure triglyceride levels^58,59^.

### Phenotypes for genetic association

In MESA, eleven phenotypes were used for genetic association study analyses: plasma LAG3 protein levels as measured by ELISA (Exam 2) and SOMAscan proteomic assay (Exams 1 and 5), HDL-C, LDL-C, triglycerides, CAC presence/absence, Agatston calcium score (among those with presence of CAC), common carotid IMT, internal carotid IMT, ABI, stroke, and MI. All of the phenotypes were examined as quantitative traits, except for CAC (presence/absence), stroke and MI which were examined as dichotomous traits. We performed inverse normal transformations for LAG3 protein levels, and performed natural-log transformation for Agatston calcium score, common carotid IMT, and internal carotid IMT due to non-normal distribution of these outcome traits. Genetic analysis of clinical events in MESA was carried out based on MESA adjudication through the year 2017.

### Genetic association analysis in MESA

We approached discovery for genetic association analyses in MESA in two ways (Fig. 1). First, we stratified data by race/ethnic groups, and performed genetic association analyses in each race/ethnic group using EPACTS software for quantitative phenotypes (https://github.com/statgen/EPACTS) and SNPTEST software for dichotomous outcome traits^60^. Quantitative trait analysis was conducted including all participants with available genotype and phenotype data, and family structure was accounted using linear mixed models in EPACTS, while the dichotomous trait analysis was conducted for a subset of participants with first-degree relatives removed. We then meta-analyzed the genetic association results across all four race/ethnic groups by fixed effects model using METAL^61^. Second, we combined all four MESA race/ethnic groups into one simple pooling group without weighting and performed genetic association analyses using EPACTS and SNPTEST. Analyses were conducted across MESA Exams 1, 2 and 5 due to the availability of LAG3 protein measurements at these examination times (Table 1).

The association of genetic variants with eleven phenotypes was conducted with adjustment for some of the following covariates: age, sex, study site, principal components (PCs) of ancestry (2 PCs for White, 1 PC for Chinese, 1 for African-American, and 3 for Hispanic, and 5 PCs for race/ethnic pooled analyses), self-reported race/ethnicity (pooled-group analysis only), HDL-C, LDL-C, triglycerides, body mass index (BMI), fasting glucose, SBP, diastolic blood pressure (DBP), current smoking, former smoking, and lipid medication use. Covariate adjustment for all lipid variables (HDL-C, LDL-C, and triglycerides) was omitted in the analysis of these three traits as phenotypic outcomes.

In genetic association analysis, we excluded variants with heterozygosity count (HC) less than 30 overall (for quantitative traits) or HC less than 30 among cases (for dichotomous traits). We then applied the false discovery rate (FDR) method to control for inflation of the type I errors due to multiple comparisons. Variants with FDR < 0.05 were considered statistically significant in this study.

### Validation study

We performed validation analyses for genetic association analysis of LAG3 protein levels in two ways (Fig. 1). First, we selected significant variants from genetic association analysis/meta-analysis results of MESA (discovery) and investigated the association of these variants in FHS (validation) that mainly focuses on white individuals of European ancestry. Second, we selected significant variants from FHS (discovery) and verified if they were also significant in MESA (validation). Genetic association analysis in FHS followed the same approach as that used for pooled analysis in MESA, with the exception that analysis in FHS did not require covariate adjustment for race/ethnicity and study site; as noted for MESA, the linear mixed models implemented in EPACTS do account for family relationships that are present in the FHS data.

### LAG3 protein association analysis

To identify proteins associated with LAG3, we performed linear regression analysis using inverse normal transformed protein levels (in MESA) and log-transformed protein levels (in FHS) for LAG3 levels as an independent variable and the individual proteins as dependent variables. These regression analyses were carried out separately in MESA Exam 1, MESA Exam 5, and FHS Offspring Exam 5 using SOMAscan proteomic measurements. In MESA, regression analyses were carried out for an unrelated subset of participants using linear regression, while in FHS related individuals were included and familial relationship adjusted using linear mixed models. LAG3 protein association analysis included covariate adjustment for age, sex, study site (in MESA), race/ethnicity (in MESA), PCs of ancestry, pack-years of smoking (in MESA), current smoking (in FHS), lipid medication, plate ID and batch (in FHS). In FHS, regression analyses did not require adjustment for race/ethnicity and study site as all of the FHS participants were white and from a single study site. In MESA, all proteomics tests were carried out in a single batch, so analyses did not require adjustment for batch. Independent discovery and validation of LAG3-associated proteins in MESA and FHS proceeded following the approach described for genetic association analysis of LAG3 protein levels (Fig. 1).

For a set of 183 LAG3-associated proteins identified by discovery and supported by validation in both MESA and FHS, we further carried out regression analyses to examine their association with HDL-C, CHD and all-cause mortality. Protein association was conducted using adjudicated events in MESA through the year 2017. In MESA, we examined associations with inverse normal transformed protein levels, while in FHS, we used log-transformed protein levels. Association of HDL-C with protein levels was examined by linear regression with covariate adjustment for age, sex, study site (in MESA), race/ethnicity (in MESA), PCs of ancestry, BMI, triglycerides, pack-years of smoking (in MESA), current smoking (in FHS), current alcohol use, LAG3 protein level, plate ID and batch (in FHS). Association of baseline protein levels with CHD and all-cause mortality was examined under a Cox proportional hazards model with covariate adjustment for age, sex, race/ethnicity (in MESA), PCs of ancestry, BMI, total cholesterol, HDL-C, SBP, DBP, pack-years of smoking (in MESA) and current smoking status (in FHS) and batch (in FHS).

### Chromatin capture analysis

We performed in situ Hi-C analysis in EBV-transformed B lymphoblasts from two female (one African-American and one Hispanic) MESA carriers homozygous for the *SCARB1* rs10846744 reference G and two female (one African-American and one Hispanic) MESA carriers homozygous for the effect C alleles. The in situ Hi-C analysis was performed as recommended by the 4D Nucleome Consortium using the four base pair cutter *DpnII* restriction enzyme and high read depth next gen sequencing (NGS) to maximize resolution of the high frequency interactions between chromatin contacts (https://www.4dnucleome.org/protocols.html). Each cell library underwent deep NGS at read depths between 1.4–3.3 billion and this was done independently twice as technical replicates for each cell library. Bioinformatic analysis was conducted using Hi-C Pro software with binning of the matrix at different resolutions and iterative correction and eigenvector decompensation normalization of the matrix for each of the four libraries^62,63^. Readouts were all valid paired-end reads and corresponding high frequency contact interaction scores. We first used HiCcompare to evaluate the quality of the technical replicates of each library^50^. We then used data generated from the million binning resolution and filtered it based on the *LAG3* chromosomal coordinates (chr12:677250–6778455) using human assembly GRCh38/hg38 (https://genome.ucsc.edu/cgi-bin/hgGateway), which yielded both *cis* (chr12) and *trans* interactions. We set interaction scores at the *LAG3* locus arbitrarily at 1 and then compared interaction scores from direct and indirect *cis* and *trans* interactions. Student *t*-test was performed with *P* ≤ 0.05 considered statistically significant.

### GeneAnalytics LAG3 protein network analysis

We inputted the protein list generated from the LAG3 protein association analyses into GeneAnalytics (GA) (https://ga.genecards.org/#input). Based on GA proprietary algorithms, scores that were identified as high (adjusted *p* ≤ 0.0001) were analyzed for the LAG3 protein network analysis.

### Statistics and reproducibility

The sample sizes for the MESA population was 3867 and for FHS study it was 1913. Multivariate linear regression analyses were performed using fully adjusted models and FDR ≤ 0.05 was considered significant. For the HiC chromatin capture assays, two-sided Student’s *t*-test was performed and *P* ≤ 0.05 was considered significant. Statistical software programs used were JMP v15 or R.

### Reporting summary

Further information on research design is available in the Nature Research Reporting Summary linked to this article.

## Supplementary information

Supplementary Information (new)

Description of Additional Supplementary Files

Supplementary Data 1-15

NR Reporting Summary

## Data Availability

Data access for MESA (phs001416) and FHS (phs000974) was approved by the TOPMed Publications & Presentations Steering Committees with data access provided by an approved project (#21279) through a database of Genotypes and Phenotypes (dbGaP) application. Hi-C chromatin capture data are available from dbGAP by using MESA phs000209 as the identifier. Interested researchers would have to apply to TOPMed and dbGaP in order to obtain access to these datasets. Source data underlying Fig. 3a, b are presented in Supplementary Data 14–15, respectively.
