# Supplementary material for: Lymphocyte activation gene-3-associated protein networks are associated with HDL-cholesterol and mortality in the Trans-omics for Precision Medicine program
Source: Commun Biol. 2022 May 2;5:362. doi: 10.1038/s42003-022-03304-0 (PMC9061762; doi:10.1038/s42003-022-03304-0)

Supplementary Figure 1:

Regional association plot for statistically significant genetic association based on meta-analysis results of MESA for ankle brachial index at Exam 5.

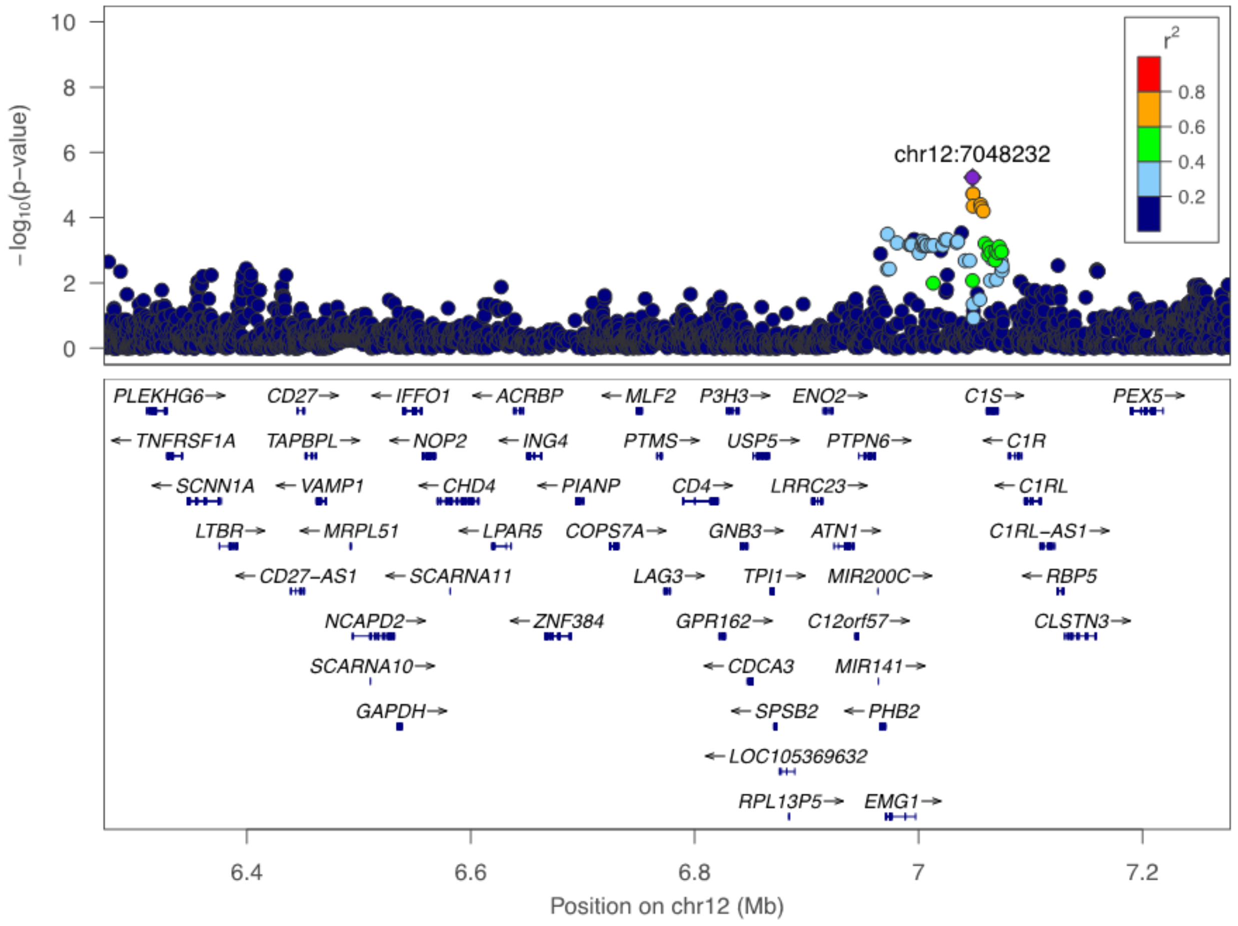

Supplement: Supplementary file 1 — Supplementary Information (new) [file 42003_2022_3304_MOESM1_ESM.pdf]
