# Supplementary material for: Lymphocyte activation gene-3-associated protein networks are associated with HDL-cholesterol and mortality in the Trans-omics for Precision Medicine program
Source: Commun Biol. 2022 May 2;5:362. doi: 10.1038/s42003-022-03304-0 (PMC9061762; doi:10.1038/s42003-022-03304-0)
Supplement: Supplementary file 2 — Description of Additional Supplementary Files [file 42003_2022_3304_MOESM2_ESM.pdf]

# Description of Additional Supplementary Files

**File name:** Supplementary Data 1
**Description:** rs3782735 association with selected outcome traits based on meta-analysis results in MESA

**File name:** Supplementary Data 2
**Description:** Summary of co-expression with LAG3 for proteins identified at FDR < 0.05 at both MESA Exams 1 and 5

**File name:** Supplementary Data 3
**Description:** Summary of co-expression with LAG3 in FHS for proteins identified at FDR < 0.05 at both MESA Exams 1 and 5 and demonstrating Bonferroni-corrected statistical significance in Framingham (P<0.05/603)

**File name:** Supplementary Data 4
**Description:** Summary of co-expression with LAG3 for proteins identified at FDR < 0.05 in FHS

**File name:** Supplementary Data 5
**Description:** Summary of co-expression with LAG3 at MESA Exams 1 and 5 for proteins identified at FDR < 0.05 in FHS and demonstrating Bonferroni-corrected statistical significance in both MESA Exams 1 and 5 (both P<0.05/657)

**File name:** Supplementary Data 6
**Description:** 183 LAG3 associated proteins that overlapped among those discovered in MESA Exams 1 and 5 and validated in FHS, and discovered in FHS and validated in MESA Exams 1 and 5

**File name:** Supplementary Data 7
**Description:** GeneAnalytics Pathway analysis

**File name:** Supplementary Data 8
**Description:** Summary of association with HDL-C in MESA for LAG-associated proteins demonstrating FDR<0.05 at both MESA Exams 1 and 5

**File name:** Supplementary Data 9
**Description:** Summary of association with HDL-C in FHS for LAG3-associated proteins discovered in MESA Exams 1 and 5 and exhibiting Bonferroni corrected statistical significance in FHS (P<0.05/13)

**File name:** Supplementary Data 10
**Description:** Summary of association with HDL-C in FHS for LAG3-associated proteins demonstrating FDR<0.05

**File name:** Supplementary Data 11
**Description:** Summary of association with HDL-C for LAG3-associated proteins discovered in FHS and demonstrating Bonferroni corrected statistical significance at both MESA Exams 1 and 5 (P<0.05/88)

**File name:** Supplementary Data 12
**Description:** Summary of association with all-cause mortality in FHS for LAG3-associated proteins discovered at FDR<0.05

**File name:** Supplementary Data 13
**Description:** Summary of association with all-cause mortality in MESA for LAG3-associated proteins discovered in FHS and exhibiting nominal associations in MESA (at P<0.05)

**File name:** Supplementary Data 14
**Description:** Supplementary Source Data for Figure 3a

**File name:** Supplementary Data 15
**Description:** Supplementary Source data for Figure 3b
