# Supplementary material for: Lymphocyte activation gene-3-associated protein networks are associated with HDL-cholesterol and mortality in the Trans-omics for Precision Medicine program
Source: Commun Biol. 2022 May 2;5:362. doi: 10.1038/s42003-022-03304-0 (PMC9061762; doi:10.1038/s42003-022-03304-0)
Supplement: Supplementary file 4 — NR Reporting Summary [file 42003_2022_3304_MOESM4_ESM.pdf]

nature portfolio

Corresponding author(s): Annabelle Rodriguez

Last updated by author(s): Jan 13, 2022

Reporting Summary

Nature Portfolio wishes to improve the reproducibility of the work that we publish. This form provides structure for consistency and transparency in reporting. For further information on Nature Portfolio policies, see our Editorial Policies and the Editorial Policy Checklist.

Statistics

For all statistical analyses, confirm that the following items are present in the figure legend, table legend, main text, or Methods section.

n/a	Confirmed	
☐	☒	The exact sample size (*n*) for each experimental group/condition, given as a discrete number and unit of measurement
☐	☒	A statement on whether measurements were taken from distinct samples or whether the same sample was measured repeatedly
☐	☒	The statistical test(s) used AND whether they are one- or two-sided<br>*Only common tests should be described solely by name; describe more complex techniques in the Methods section.*
☐	☒	A description of all covariates tested
☐	☒	A description of any assumptions or corrections, such as tests of normality and adjustment for multiple comparisons
☐	☒	A full description of the statistical parameters including central tendency (e.g. means) or other basic estimates (e.g. regression coefficient) AND variation (e.g. standard deviation) or associated estimates of uncertainty (e.g. confidence intervals)
☐	☒	For null hypothesis testing, the test statistic (e.g. *F*, *t*, *r*) with confidence intervals, effect sizes, degrees of freedom and *P* value noted<br>*Give P values as exact values whenever suitable.*
☒	☐	For Bayesian analysis, information on the choice of priors and Markov chain Monte Carlo settings
☒	☐	For hierarchical and complex designs, identification of the appropriate level for tests and full reporting of outcomes
☐	☒	Estimates of effect sizes (e.g. Cohen's *d*, Pearson's *r*), indicating how they were calculated

Our web collection on statistics for biologists contains articles on many of the points above.

Software and code

Policy information about availability of computer code

Data collection	No custom software or code was used to collect data for the current investigation. TOPMed Freeze 8 whole genome sequencing (WGS) data for MESA and Framingham were processed as described in Taliun et al. (PMID: 33568819) and received from the TOPMed Data Coordinating Center. Proteomic data were collected using the Somascan platform. All MESA samples were profiled with Version 1.3k. FHS samples were either profiled with Version 1.1k with 1,124 aptamers or with Version 1.3k with 1,305 aptamers.
Data analysis	JMP version 15 and R version 3.6

For manuscripts utilizing custom algorithms or software that are central to the research but not yet described in published literature, software must be made available to editors and reviewers. We strongly encourage code deposition in a community repository (e.g. GitHub). See the Nature Portfolio guidelines for submitting code & software for further information.

Data

Policy information about availability of data

All manuscripts must include a data availability statement. This statement should provide the following information, where applicable:

- Accession codes, unique identifiers, or web links for publicly available datasets
- A description of any restrictions on data availability
- For clinical datasets or third party data, please ensure that the statement adheres to our policy

Individual whole-genome sequence data for TOPMed whole genomes are available through dbGaP. The dbGaP accession numbers are: Framingham Heart Study (FHS) phs000974, and Multi-Ethnic Study of Atherosclerosis (MESA) phs001416. Data in dbGaP can be downloaded by controlled access with an approved application submitted through their website: https://www.ncbi.nlm.nih.gov/gap.

Field-specific reporting

Please select the one below that is the best fit for your research. If you are not sure, read the appropriate sections before making your selection.

☒ Life sciences ☐ Behavioural & social sciences ☐ Ecological, evolutionary & environmental sciences

For a reference copy of the document with all sections, see nature.com/documents/nr-reporting-summary-flat.pdf

Life sciences study design

All studies must disclose on these points even when the disclosure is negative.

Sample size	Genetic analyses were conducted for participants from MESA (n=3867) and FHS (n=1913). Proteomic analyses were carried out in MESA Exam 1 (n=938), MESA Exam 5 (n=929), and FHS (n=1913).
Data exclusions	Missing genetic data (for genetic analyses) or proteomic data (for proteomic analyses). MIssing phenotypes or covariates. Covariates included in analyses included age, sex, study site, principal components (PCs) of ancestry (2 PCs for White, 1 PC for Chinese, 1 for African-American, and 3 for Hispanic, and 5 PCs for race/ethnic pooled analyses), self-reported race/ethnicity (pooled-group analysis only), HDL-C, LDL-C, triglycerides, body mass index (BMI), fasting glucose, SBP, diastolic blood pressure (DBP), current smoking, former smoking, and lipid medication use, as detailed for specific models in the text.
Replication	Genetic association resutls that met multiple comparisons correction (FDR<0.05) in MESA were carried forward for validation in FHS. Although we had planned to carry forward genetic discoveries from FHS for validation in MESA, there were no genetic associations meeting FDR<0.05. For proteomic analyses, we also discovered associations at FDR<0..05 in MESA and carried those forward for validation in FHS. Similarly FDR<0.05 discoveries from FHS were carried forward for validation in MESA.
Randomization	N/A
Blinding	N/A

Reporting for specific materials, systems and methods

We require information from authors about some types of materials, experimental systems and methods used in many studies. Here, indicate whether each material, system or method listed is relevant to your study. If you are not sure if a list item applies to your research, read the appropriate section before selecting a response.

Materials & experimental systems

n/a	Involved in the study	
☒	☐	Antibodies
☐	☒	Eukaryotic cell lines
☒	☐	Palaeontology and archaeology
☒	☐	Animals and other organisms
☐	☒	Human research participants
☒	☐	Clinical data
☒	☐	Dual use research of concern

Methods

n/a	Involved in the study	
☒	☐	ChIP-seq
☒	☐	Flow cytometry
☒	☐	MRI-based neuroimaging

Eukaryotic cell lines

Policy information about cell lines

Cell line source(s)	Epstein-Barr virus transformed human B lymphoblasts
Authentication	Genotyping was confirmed in the laboratory of one of the authors
Mycoplasma contamination	Mycoplasma contamination was not confirmed
Commonly misidentified lines (See ICLAC register)	Not applicable

Human research participants

Policy information about studies involving human research participants

Population characteristics	The median baseline age of participants from MESA (Exam 1) was 61 years (with an interquartile range of 53.0 - 69.0 years); 50.7 % were women; and self-reported race/ethnic distribution was 41.5% White, 23.2% Hispanic, 22.8% African-American,

	and 12.5% Chinese-American. In FHS (Offspring Exam 5), the median age of participants was 55 years (with an interquartile range of 47-63 years); 53.5% were women; and were 100% White participants.
Recruitment	MESA is a longitudinal study of subclinical CVD and risk factors that predict progression to clinically overt CVD or progression of the subclinical disease. The first clinic visits (Exam 1) occurred from 2000 to 2002 in 6,814 participants recruited from 6 field centers across the United States, and all participants were free of clinical CVD at baseline. The self-reported ancestry distribution is approximately 38% White, 28% African-American, 22% Hispanic, and 12% Asian (predominantly of Chinese descent). MESA has been enhanced by many ancillary studies focused on specific phenotypic and exposure domains. One ancillary study (MESA Family Study) exclusively recruited African-American and Hispanic family members specifically for genetic studies. In contrast, the FHS is a single community-based cohort initiated in 1948. Three generations of participants have been recruited, and the majority of them were white individuals of European ancestry. Participants were invited to attend physical examinations every 2-8 years. The current study was restricted to the Offspring cohort participants who attended their fifth clinical examination cycle during 1991-1995.
Ethics oversight	All study participants provided written informed consent. This research was reviewed and approved by the Institutional Review Boards at all recruitment sites, as well as at the Universities carrying out hands on research for the current student, namely the University of Virginia, Boston University and the University of Connecticut.

Note that full information on the approval of the study protocol must also be provided in the manuscript.
